# Ceramides in Parkinson’s Disease: From Recent Evidence to New Hypotheses

**DOI:** 10.3389/fnins.2019.00330

**Published:** 2019-04-02

**Authors:** Nicoletta Plotegher, Luigi Bubacco, Elisa Greggio, Laura Civiero

**Affiliations:** Laboratory of Cellular Physiology and Molecular Biophysics, Department of Biology, University of Padua, Padua, Italy

**Keywords:** ceramides, Parkinson’s disease, glucocerebrosidase, insulin, neuroinflammation, lipid rafts, Gaucher disease

## Abstract

Ceramides (Cer) constitute a class of lipids present in the cell membranes where they act as structural components, but they can also work as signaling molecules. Increasing genetic and biochemical evidence supports a link between deregulation of ceramide metabolism in the brain and neurodegeneration. Here, we provide an overview of the genes and cellular pathways that link Cer with Parkinson’s disease and discuss how ceramide pathobiology is gaining increasing interest in the understanding of the pathological mechanisms that contribute to the disease and in the clinical and therapeutic side.

## Introduction

Ceramides (Cer) belong to the class of sphingolipids, ubiquitous lipids constituted by a sphingosine moiety and a fatty acid. They are essential structural elements of the lipid bilayer of cell membranes, however, a number of previously unrecognized roles in the regulation of a variety of cellular process have also been described.

**FIGURE 1:**
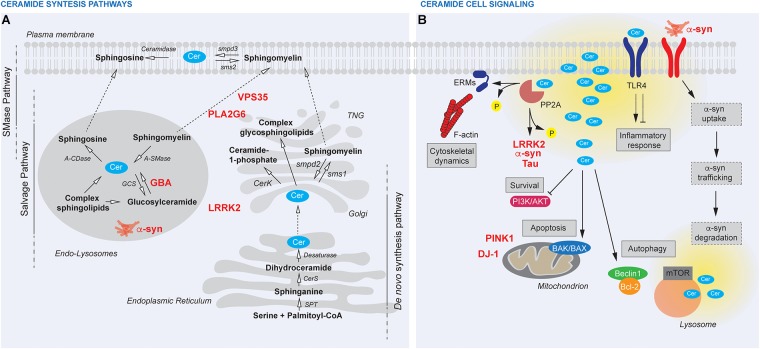
**(A)** Ceramide (Cer) synthesis pathways and their cellular compartmentalization. The SMase pathway takes place at the plasma membrane, where sphingomyelin is hydrolyzed to Cer by smpd3 and reconverted back by sphingomyelin synthase (sms2). The *de novo* synthesis sits at the endoplasmic reticulum, where Cer is synthesized starting from the condensation of palmitoyl-CoA and serine by serine palmitoyl-CoA acyltransferase (SPT) and then by the action of dihydrosphingosine synthase (CerS) and desaturase. Cer are further processed by sphingomyelin synthase (sms1) and smpd2 or phosphorylated to Cer-1-phopshate by (CerK) at the *trans*-Golgi network (TGN). Within the endolysosomal route, a number of enzymes, namely SMases (acid SMase, A-SMase), cerebrosidases (acid b-glucosidase, GBA), ceramidases (acid ceramidase, A-CDase), and Cer synthases (glycosyl synthase, GCS), are involved in the recycling of sphingosine. **(B)** Cer cell signaling. Cer are bioactive lipids upstream several pathways and local or global dysregulation of Cer amount (yellow) can affect cellular biology at multiple levels thus contributing to PD pathogenesis. Cer directly activate PP2A thus inducing cytoskeletal dynamics through ERMs dephosphorylation or regulating PD-linked proteins (LRRK2, a-syn, Tau). Cer inhibit cell survival through PI3K/AKT and induce apoptosis via BAK/BAX. Cer affect also the autophagic flux via Beclin1/Bcl-2 or mTOR. Changes in membrane fluidity, membrane trafficking, lysosomal functionality induced by Cer unbalance possibly interfere with a-syn up-take and degradation. TLR4 receptor modulation by Cer impacts inflammatory response, Cer synthesis itself and the whole body metabolism (pink boxes).

Three major pathways are responsible for Cer synthesis: (1) the sphingomyelinase (SMase) pathway, where sphingomyelin is hydrolyzed to Cer; (2) the *de novo* synthesis, where Cer is synthesized through a four-step biochemical pathway starting from the condensation of palmitoyl-CoA and serine; (3) the salvage pathway, where a number of enzymes, namely SMases, cerebrosidases, ceramidases, and ceramide synthases, are involved in the recycling of sphingosine (Figure 1; Kitatani et al., 2008). Cer is a central lipid in the sphingolipids metabolism since all sphingolipids are synthesized starting from Cer and are hydrolyzed back to Cer (Kitatani et al., 2008).

In the brain, Cer play a variety of functions to coordinate brain homeostasis. We will not review here the literature discussing the role of Cer in brain physiology, for which the reader can refer to some other reviews (Mencarelli and Martinez-Martinez, 2013; Cruciani-Guglielmacci et al., 2017). However, it is worth pointing that the brain is the organ with the highest proportion of lipid content, both in neurons, and glial cells (especially oligodendrocytes). The neuronal plasma membrane is enriched in lipid rafts, which are themselves rich in Cer (Mencarelli and Martinez-Martinez, 2013). Lipid rafts are specialized membrane microdomains that enable the compartmentalization of a variety of cellular processes by acting as hubs for the assembly of signaling complexes (Daniel and Kai, 2010). Neuronal lipid rafts, in addition to influencing membrane fluidity, play a crucial role in orchestrating synaptic neurotransmission and receptor, clustering, and trafficking (Sebastião et al., 2013). Aging brains display increased Cer content (Cutler et al., 2004) and accumulation of Cer in neuronal rafts is associated with impaired receptor trafficking and synapse loss (Hering et al., 2003). Lipid buildup due to impaired lysosomal degradative capacity of neurons or glia cells results in severe metabolic storage disorders collectively named lysosomal storage diseases (LSDs). Interestingly, homozygous mutations in glucocerebrosidase (GBA), a lysosomal enzyme breaking down glucosylceramide (GlcCer) into glucose and Cer, cause a LSD known as Gaucher disease (GD), while the same mutations in heterozygosis increase by five folds the lifetime risk of Parkinson’s disease (PD) (Sidransky et al., 2009). PD is a neurodegenerative disorder characterized by the preferential death of dopaminergic neurons in the *substantia nigra pars compacta* (SNpc) and the accumulation into Lewy bodies of the presynaptic protein alpha-synuclein (α-syn) within surviving neurons (Spillantini et al., 1997). Mounting evidence link several PD-associated genes and molecular pathways to Cer biology. In this review we critically discuss how deregulation of Cer synthesis/metabolism may result in brain dyshomeostasis and, in turn, in PD-associated neurodegeneration.

## Ceramides and Parkinson’s Disease: Genetic Evidence

Sidransky et al. (2009) observed a strong association between *GBA* mutations and PD, confirming earlier observations in small cohorts of patients (Tayebi et al., 2001; Aharon-Peretz et al., 2004; Lwin et al., 2004). While it is not clear whether heterozygous mutations in *GBA* increase PD risk through accumulation of GlcCer or whether other mechanisms are triggered by mutated enzyme, several studies observed that PD heterozygous mutations in *GBA* result in α-syn buildup in neurons (Taguchi et al., 2017; Yun et al., 2018), likely because mutant GBA negatively impacts on the degradative capacity of the neuron. We recently observed that mice knockout for another autosomal dominant PD-associated protein, namely leucine rich repeat kinase 2 (LRRK2), display altered sphingolipid composition and increased Cer levels in the brain, which paralleled with alterations in GBA expression (Ferrazza et al., 2016). While the exact molecular mechanisms underlying this GBA-LRRK2 relationship are unresolved, we can speculate that mutant LRRK2 may result in impaired levels of Cer by deregulating membrane trafficking, which in turn affect the autophagic-lysosomal pathway, a process robustly associated with LRRK2 function (Roosen and Cookson, 2016). Additional genetic evidence supports a link between unbalanced Cer levels and PD. Genetic ablation of PLA2G6, a protein mutated in recessive forms of parkinsonism, was shown to impair store-operated Ca^2+^ signaling, with consequent autophagic dysfunction and loss of dopaminergic neurons in PD patient-derived cells and transgenic mice (Zhou et al., 2016). Considering that alterations in Ca^2+^ homeostasis result in accumulation of Cer (Jayadev et al., 2017), we can speculate that PLA2G6 controls Cer balance by orchestrating calcium homeostasis in the intracellular stores. Further supporting a link between PLA2G6 and Cer, Lin et al. (2018) recently observed that loss of PLA2G6 results in accumulation of Cer in *Drosophila melanogaster*, and neuronal cells. Mechanistically, they propose that loss of PLA2G6 increases Cer content by interfering with the retromer function. Of note, PLA2G6 binds and stabilizes the retromer subunit VPS35, a protein that is also mutated in recessive PD (Follett et al., 2013). Overexpression of α-syn itself impairs retromer function, causing Cer buildup with impact on lysosomal activity (Lin et al., 2018). Finally, the PD VPS35 D620N mutation enhances LRRK2 kinase activity toward its endogenous substrate RAB10 and VPS35 is required for LRRK2 kinase activity (Mir et al., 2018). All these findings support the existence of a tight connection among PD genes acting at the Golgi-endosome-lysosome interface and Cer content (Figure 1). Importantly, elevated Cer levels modeled in genetic-based preclinical systems can be recapitulated in PD patients, in which plasma levels of certain sphingolipids are found to be altered (Mielke et al., 2013; Guedes et al., 2017). These findings implicate that dosing Cer levels in PD patients may serve as a valuable disease biomarker (Kurz et al., 2018).

## Role of Ceramides in Shaping the Structure of Membranes

While accumulating evidence links impaired Cer content with PD, it is unclear whether their physical or their chemical (or both) properties underlie the observed neuronal toxicity.

A number of studies support the concept that Cer and GlcCer define some of the biophysical properties of membranes and their functional plasticity (Mencarelli and Martinez-Martinez, 2013; Futerman and Hardy, 2016). Cer and GlcCer content is, at least in part, regulated by the opposite actions of GBA and glucosylceramide synthase (GCS) (Figure 1). In spite of the significant chemical difference between Cer and GlcCer, i.e., the presence of the glucose moiety in the C1 position of Cer, their functional impact is similar (Varela et al., 2013, 2017). In fact, they both affect the properties of fluid model membranes and they are both able to form tightly packed gel domains. However, Cer and GlcCer differ in their capacity to promote changes in the membrane shape. To this regard, Varela et al. (2017) recently reported that GlcCer-induced membrane perturbation is pH dependent. This observation hints to the possible impact of Cer:GlcCer ratio in defining the number and shape of lysosomes, with the caveat that the asymmetric membrane composition may add a further layer of complexity. Interestingly, the authors observed decreased membrane fluidity in cells treated with the GBA inhibitor conduritol B epoxide and in GD patient derived cells (Batta et al., 2018; Pavićević et al., 2018). In addition to the GlcCer:Cer ratio, it is also important to consider the membrane fraction of cholesterol present. The cholesterol interaction with both Cer (Artetxe et al., 2017) and GlcCer (Varela et al., 2017) has been proved to affect the membrane domain phase transition likely in synergy with the ratio of the diverse Cer present, which, at least in part, depends on the level of GBA activity. Thus, reduced GBA activity due to PD mutations is predicted to affect membrane fluidity by interfering with GlcCer content and cholesterol interaction and we can speculate that the lysosome will be particularly sensitive to GlcCer levels.

It is worth mentioning that also the length of the acyl chain and the saturation of Cer molecules can impact on membrane properties (Castro et al., 2014). Accordingly, the levels of different Cer species were shown to be differentially altered in PD brains (Abbott et al., 2014). However, it is unclear at this stage how Cer may impact membrane properties mechanistically, as well as the patho-physiological consequences for the neurons of these overall changes.

## Ceramides as Signaling Molecules

As mentioned, Cer are structural components of the cell membrane with important roles in maintaining barrier function and fluidity. However, Cer-related changes in membrane structure and the direct binding of Cer to target molecules generate specific cell signals. For this reason, Cer, its derivative spingosine-1-phosphate (S1P) and other sphingolipids named “bioactive lipids,” function as second messengers. They intervene in a variety of cellular pathways including regulation of cell growth, death, adhesion, migration, inflammation, and intracellular trafficking. Enzymes of lipid metabolism are interconnected and work as a network by adjusting sphingolipid conversion upon specific stimuli. Also, the action of bioactive lipids is not restricted to microdomain and can spread through diffusion within different membrane compartments. Therefore, sphingolipid signaling manifests additional layers of complexity compared with the canonical signaling cascade complicating the understanding of their role in specific pathways relevant for PD.

Several neurodegenerative diseases are accompanied by alterations in Cer as well as S1P composition [reviewed in (Wang and Bieberich, 2018)]. An imbalance in Cer level and Cer species is a common feature in PD mouse models and PD *post mortem* brains (Abbott et al., 2014; Murphy et al., 2014; Ferrazza et al., 2016) and has been hypothesized as an upstream event responsible for neuronal degeneration through various pathological processes including apoptosis, autophagy, inflammation, and phagocytosis.

Ceramides directly activate apoptosis via different mechanism depending on cell types including the induction of DNA fragmentation (Obeid et al., 1993), the formation of Cer channels in the mitochondria outer membrane favoring BAK/BAX activation (Chipuk et al., 2012), and the regulation of caspase 3 by their compartmentalization in the late endosome organelles (Blom et al., 2015). In addition, Cer-induced death of dopaminergic cells has been described in association with early inhibition of the neuronal survival PI3K/AKT pathway (Arboleda et al., 2010). Activation of pro-apoptotic cascades together with inhibition of anti-apoptotic pathways contributes to alterations in mitochondrial homeostasis, a common feature in both genetic, and sporadic PD. Of note, overexpression of two proteins involved in recessive monogenic PD and important for mitochondria quality control, PINK1 and DJ-1, confers a neuroprotective effect against the exposure of C2 ceramide through the activation of PI3K/AKT pathway (Sánchez-Mora et al., 2012; Jaramillo-Gómez et al., 2015; Figure 1). Although no reports are available on differential S1P levels in PD patients versus control, modulation of S1P signaling pathway shows neuroprotective effects in PD mouse models (Zhao et al., 2017). These findings are in agreement with the role of S1P in stimulating cell survival and proliferation.

Sphingolipid metabolites are also implicated in the regulation of autophagy (Young et al., 2013). Similar to amino acid starvation, Cer triggers autophagy by interfering with the mTOR-signaling pathway, and by dissociating the Beclin1/Bcl-2 complex. In *Drosophila*, neurodegeneration caused by impairment in the autophagic flux depend on an elevation in total Cer levels. Specifically, degeneration is ameliorated when the pool of available Cer is further increased, and it is exacerbated when Cer levels decrease by altering sphingolipid catabolism or blocking *de novo* synthesis (Hebbar et al., 2015).

One of the immediate downstream targets of Cer is the protein phosphatase 2A (PP2A) (Mukhopadhyay et al., 2009). Interestingly, LRRK2, α-syn, and tau are all PP2A-target proteins, and their phosphorylation patterns are linked to disease (Taymans and Baekelandt, 2014). Therefore, dysregulation of Cer metabolism could switch on/off specific cell programs related to PD, although no studies are available on the precise molecular mechanism that connects Cer unbalance and PP2A enzymatic action on LRRK2, α-syn, and tau.

The Cer-PP2A axis is also key in the regulation of cytoskeletal dynamics, an important aspect PD etiopathogenesis. Specifically, Cer contribute to erzin, razin, and moesin (ERM) dephosphorylation via PP2A causing their detachment from the cell membrane [reviewed in (Adada et al., 2014)]. Accordingly, accumulation of GlcCer in *Gba2^-/-^* mice augments actin polymerization and promotes microtubules persistence, resulting in a higher number of filopodia and lamellipodia, and longer microtubules (Raju et al., 2015). Therefore, Cer-mediated cytoskeleton deficits could disturb neuronal arborization, transport along cell processes, neuro-, and gliotrasmitter release as well as microglia activation (Figure 1).

Growing evidence suggests that Cer plays a pro-inflammatory role. Since inflammation within the central nervous system (CNS) is a major component in PD, unraveling the signaling modulation evoked by Cer in microglia is particularly relevant in the field. Interestingly, Cer help NLRP3 inflammasome assembly with a consequent cytokine release (Scheiblich et al., 2017). Moreover, Cer have been reported to promote stabilization of Toll-like receptor 4 (TLR4) and, therefore, enhance LPS-induced pro-inflammatory signaling (Płóciennikowska et al., 2015). Conversely, one study suggests that exogenous C2 ceramide inhibits TLR4 signaling by interfering with LPS and TLR4 interactions (Jung et al., 2013). In the CNS, TLRs are expressed in glial cells and upregulated in PD brains (Dzamko et al., 2017; Mariucci et al., 2018). Both TLR4 and TLR2 are found to trigger neuroinflammation upon α-syn activation (Noelker et al., 2013; Dzamko et al., 2017; Figure 1). Therefore, a more detailed understanding of the implication of Cer dysregulation in α-syn-mediated glia activation would be desirable.

Recently, several enzymes (ELOVL1, CERS2, and SGMS1) that participate in consecutive steps of the Cer biosynthetic pathway were identified in a genome-wide screen as required for phagocytosis (Haney et al., 2018). Glia are emerging as phagocytic cells involved in α-syn clearance in the CNS (Janda et al., 2018; Jung and Chung, 2018) and impaired phagocytic clearance appears to be implicated in many neurodegenerative disorders. Therefore, mutations in PD-linked genes that impact Cer metabolism might compromise phagocytic clearance at multiple levels thus participating in PD pathogenesis. On one hand, membrane distribution of receptors (e.g., receptors involved in α-syn uptake) might be affected by Cer unbalance. On the other hand, lysosome impairment caused by alteration of Cer:GlcCer ratio (e.g., GBA mutations) or endo-lysosomal membrane trafficking defects induced by accumulation of Cer (e.g., LRRK2, VPS35, and PLA2G6 mutations) could contribute to α-syn accumulation in glial cells.

## Role of Ceramides in the Cellular Metabolism of the Brain and Implications for PD

If Cer and GlcCer have a strong impact on cellular and whole-body metabolism, metabolism can also impact the activity of Cer enzymes and Cer biosynthetic pathways. For example, the *de novo* pathway of Cer can be regulated by various signals. In particular, the availability of the initial substrates for the synthesis, i.e., palmitate and serine, hormone signals, and inflammatory agonists can be all drivers of Cer synthesis or degradation. Among the others, it was found that TLR4 agonists promote Cer synthesis, TLR4 can induce transcriptional regulation of Cer synthesis and the lack of TLR4 cause a reduced ability to accumulate Cer (Sims et al., 2010; Holland et al., 2011). In the frame of PD, TLR4 knockout animals are less vulnerable to dopamine depletion induced by MPTP treatment, at least in the striatum, suggesting a role for TLR4-related neuroinflammation in PD (Conte et al., 2017), possibly associated with a decrease in Cer levels. In addition to upregulation of TLRs in PD brains, TLR4 gene polymorphisms are associated with sporadic PD in a Chinese population (Zhao et al., 2015). Interestingly, Cer synthesis is a requisite for the TLR4-induced insulin resistance and the activation of TLR4 is able to induce insulin resistance in certain brain regions (i.e., the hypothalamus) (Holland et al., 2011). Insulin sensitivity plays also an important role in PD. Beside the fact that this mechanism is crucial for brain homeostasis (Kullmann et al., 2016), diabetic individuals show an increased risk of developing PD, and PD and diabetes etiopathogenesis seem to share some molecular mechanisms (reviewed in Santiago and Potashkin, 2013). More recently, it was also suggested that agonist of the glucagon-like peptide-1 (GLP-1) receptor, already used to induce insulin release from pancreatic cells in diabetic patients, may be a new therapeutic strategy in PD. Accordingly, GLP-1 agonists administered in PD animal models showed beneficial effects, and, the first clinical trials presented encouraging results and suggest that these drugs may be considered as promising disease-modifying agents in PD (Athauda et al., 2017).

The downstream molecular mechanisms that are altered by defective activation of TLR4 and insulin receptor are mainly the NF-kB transcription factor and the AKT signaling pathways, involved among the other mechanisms in neuroinflammation and cell survival. Interestingly, manipulation of the NF-κB pathway was already suggested to be a possible target for therapies in PD, and the chance of understanding the upstream events involved in PD pathogenesis through NF-kB is quite intriguing but still unresolved (Flood et al., 2011).

Accumulating evidence further indicates that changes in Cer levels impact on different mechanisms that regulate cellular metabolism, such as nutrient handling and mitochondrial function. Adequate metabolic dealing not only with glucose, but also with fatty acids and amino acids, is crucial for proper brain function. Interestingly, it was shown that Cer can impact insulin-stimulated glucose uptake, can have a negative effect on lipid uptake and can affect amino acid transport in different conditions and in different models (Summers et al., 1998; Hyde et al., 2004; Schwenk et al., 2010). How this may specifically contribute to neurodegeneration in PD needs further elucidation.

As previously mentioned, Cer deregulation can affect mitochondrial function. On one side it was proposed that Cer can form channels in the outer mitochondrial membranes inducing apoptotic signals (Siskind, 2005; Chipuk et al., 2012). Other evidence suggested that Cer are able to alter mitochondrial bioenergetic. For example, it was shown that Cer can inhibit electron transport complex I or complex III (Gudz et al., 1997; Di Paola et al., 2000), which in turn can lead to elevated reactive oxygen species. Moreover, oxidative phosphorylation impairment leads to a reduced ATP production, which can ultimately impact on cellular metabolism.

Interestingly, also GlcCer was shown to play a role in regulating energy storage and insulin sensitivity, and GD patients present increased insulin resistance in certain cases (Langeveld et al., 2008; Aerts et al., 2015). This suggests that both the synergic and separate effects of Cer and GlyCer species need to be carefully evaluated when attempting to understand the contributes of these bioactive lipids to metabolic defects in PD.

## Conclusion

A growing body of evidence links impaired Cer metabolism with PD. Cer are ubiquitous sphingolipids playing a variety of roles in shaping cell membranes but also as bioactive molecules able to orchestrate critical cellular processes that are deregulated in PD. Several PD-linked genes influence or are influenced by Cer levels. In addition, Cer levels are elevated in PD patients supporting the notion that deregulated Cer metabolism is a central theme in the disease. However, it still remains to be clearly proved whether unbalanced Cer levels are a cause or a consequence of disease. Future studies providing a more detailed mechanistic picture of the link between Cer and PD will be of key importance to nominate specific targets within the Cer metabolic pathways or among Cer interactors that can be tested as disease modifying therapies.

## Author Contributions

NP, LB, EG, and LC conceived, wrote, and proofread the manuscript.

## Conflict of Interest Statement

The authors declare that the research was conducted in the absence of any commercial or financial relationships that could be construed as a potential conflict of interest.
